# Evaluation of oxidative stress in an experimental model of Crohn's disease treated with hyperbaric oxygen therapy

**DOI:** 10.1016/j.clinsp.2023.100305

**Published:** 2023-11-15

**Authors:** Fernanda Serafim Nakutis, Iêda Nishitokukado, Fabiana Maria dos Santos, Carmen Lucia Ortiz-Agostinho, Daniel Teixeira de Alencar, Cassiana Ganem Achtschin, Valeria Sutti Nunes, André Zonetti Arruda Leite, Aytan Miranda Sipahi

**Affiliations:** a Laboratory of Experimental Clinical Gastroenterology (LIM-07), Division of Clinical Gastroenterology and Hepatology, Hospital das Clínicas da Faculdade de Medicina da Universidade de São Paulo (HCFMUSP), São Paulo, SP, Brazil; b Lipids Laboratory (LIM-10), Division of Endocrinology and Metabolism, Hospital das Clínicas da Faculdade de Medicina da Universidade de São Paulo (HCFMUSP), São Paulo, SP, Brazil; c Division of Clinical Gastroenterology and Hepatology, Hospital das Clínicas da Faculdade de Medicina da Universidade de São Paulo (HCFMUSP), São Paulo, SP, Brazil

**Keywords:** Inflammatory bowel disease, Experimental model, Hyperbaric oxygen therapy

## Abstract

*Introduction:* Treatments of Inflammatory Bowel Disease (IBD) are able to control symptoms in most cases, however, a fraction of patients do not improve or have a loss of response to treatments, making it important to explore new therapeutic strategies. Hyperbaric oxygen therapy (HBO) may represent one of them. The aim of this study was to evaluate the effects of HBO therapy in an experimental model of IBD.

*Methods:* Sixty male BALBc mice were divided into six groups. Group 1 was colitis-induced with trinitrobenzene sulfonic acid (TNBS) + ethanol, group 2 received TNBS + ethanol plus HBO, group 3 received only ethanol, group 4 received ethanol plus HBO, group 5 received saline solution, and group 6 received saline solution plus HBO. HBO was performed for four days, subsequently, the mice were evaluated daily. At the end of the study, samples from the intestine were collected for histological analysis as well as for measurement of antioxidant enzymes and cytokine levels.

*Results:* HBO significantly improved the clinical and histological status of the animals. Treatment with HBO increased the activity of the antioxidant enzymes superoxide dismutase (SOD) and glutathione peroxidase (GPx) in all of the groups; moreover, the difference was only significant between the TNBS and TNBS + HBO groups and treatments promoted a reduction in the proinflammatory cytokines IFN-γ, IL-12, IL-17 and TNF-α and increased the anti-inflammatory cytokines IL-4 and IL-10, with no changes in IL-13.

*Conclusion:* HBO effectively treats TNBS-induced colitis by increasing the activity of antioxidant enzymes and modulating cytokine profiles.

## Highlights

• HBO promoted a significant improvement in the clinical and histological statuses of the animals of this study experimental.
• HBO is effective for the treatment of colitis by increasing the activity of antioxidant enzymes.
• This study demonstrated a modulation of the immune response by reducing the expression of inflammatory cytokines and increasing the anti-inflammatory cytokines.

## Introduction

Inflammatory bowel disease (IBD) is defined as chronic inflammation of the gastrointestinal tract without a specific cause or pathogen and comprises two major gastrointestinal disorders: Crohn's disease (CD) and ulcerative colitis (UC).^1^ IBD results from an abnormal interaction between the intestinal microbiota and mucosal immune cells as well as genetic susceptibility and the environment.^2^, ^3^, ^4^, ^5^ Moreover, experimental models and clinical studies have indicated that reactive oxygen species (ROS) in the intestinal mucosa, derived from the activation of inflammatory cells, contribute to the pathogenesis of IBD.^6^,^7^

Current therapies used for the treatment of IBD are effective in controlling the disease course; however, approximately one-third of patients do not show improvement after therapy, in addition to a gradual loss of response to treatment,^8^,^9^ which makes it extremely important to develop new strategies for the treatment of non-responsive patients. Hyperbaric oxygen (HBO) therapy represents a new treatment option for patients with IBD, in which patients are exposed to 100% oxygen under a total pressure greater than 1 atm (ATM) total pressure.^10^, ^11^, ^12^, ^13^ The therapeutic action results from increased oxygen saturation in the blood and tissues, which produces a variety of effects, such as the synthesis of growth factors that promote neoangiogenesis, wound healing, and improvements in tissue inflammation, which persist after the removal of the patient from the chamber.^14^, ^15^, ^16^, ^17^, ^18^, ^19^, ^20^, ^21^, ^22^

In the present study, the authors evaluated the effect of HBO on oxidative stress in a trinitrobenzene sulfonic acid (TNBS) experimental model of inflammatory bowel disease and examined the inflammatory cytokine profile and activity of the antioxidant enzymes superoxide dismutase (SOD), glutathione peroxidase (GPx), and glutathione reductase (GR).

## Materials and methods

### Animals

BALB/c mice (20-25 grams) were obtained from the Animal House of the School of Medicine of the University of São Paulo (FMUSP) and maintained in cages at the Laboratory of Experimental Clinical Gastroenterology (LIM-07). The mice were fed standard mouse chow and tap water ad libitum throughout the study, following protocols approved by the Institutional Animal Care and Use Committee. The experimental protocol n° 055/12 was approved by the Ethics Committee of the FMUSP.

The groups were not randomized because the authors used animals of the same lineage. The sample size was calculated based on the law of diminishing returns. According to this method a value “E” is measured, which is nothing but the degree of freedom of analysis of variance (ANOVA). “E” can be measured by the following formula:

“E” = Total number of animals − Total number of groups

In this study, “E” = (10 × 6) - 6 = 54. As this number was greater than 20, the sample size in this experiment was adequate.

### Experimental design

Intestinal inflammation was induced using an adapted model proposed by Wirtz et al.^23^ Half a milligram of 5% 2,4,6-trinitrobenzenesulfonic acid (TNBS) was diluted in 70% ethanol 1:1 and administered via rectal instillation in previously anesthetized animals. The installation was performed only once on day 1 of the experiment.

The animals were divided into six groups (n = 10 mice/group): mice treated with TNBS + 35% ethanol (TNBS); mice treated with TBNS + 35% ethanol plus HBO (TNBS + HBO); mice treated with 35% ethanol (ETHANOL); mice treated with 35% ethanol plus HBO (ETHANOL+HBO); mice treated with saline solution (0.9% NaCl) (SALINE); and mice treated with saline solution plus HBO therapy (SALINE+HBO).

The authors used the same treatment order and measurements for all cages, which were kept in the same place to minimize potential confounders. The group allocation was not blinded. All the animals were included in the final analysis.

### Hyperbaric oxygen therapy

HBO was performed at the Laboratory of Experimental Clinical Gastroenterology (LIM-07) using an Ecobar 400 chamber (ECOTEC®). The animals in the HBO groups started treatment on day 1 with once-daily administration during the 4 days of treatment. During the sessions, the mice were exposed to 100% O_2_ for 60 min at 2 ATM with the addition of 10 min for gradual compression and decompression.^24^

### Clinical evaluation of mice

Clinical evaluation of the mice was performed daily according to the parameters and indices proposed by Scheinin et al.^25^ Values of 0 or 1 were assigned according to the following findings: excreted perianal mucus, rectal prolapse, diarrhea, or weight loss greater than 5%.

### Tissue extraction and storage of extracted tissue

The animals were sacrificed via intraperitoneal injection of a solution of ketamine and xylazine on day 4 after the last HBO session. The final portion of the proximal colon was removed; subsequently, part of the tissue was frozen in liquid nitrogen and stored at -80°C, and the other part was stored in a 10% buffered formalin solution.

### Histological evaluation of chronic inflammation

The tissue was fixed in formalin, embedded in paraffin, and sections of the colon were stained with hematoxylin and eosin to assess the degree of inflammation. The histological activity of the disease was evaluated using a protocol developed by Gulec et al.^26^ for experimental colitis.

### Tissue preparation for oxidative stress analysis

Fragments of frozen tissue were homogenized at low speed in 0.1 M potassium phosphate buffer (pH = 7.0) in a volume corresponding to 3 times the absolute sample mass and centrifuged at 1,010 xg for 20 minutes at 4°C. The supernatant was collected and centrifuged at 11,200 xg for 20 minutes at 4°C and then mixed with 5 mL of 0.1 M potassium phosphate buffer (pH = 7.0) and centrifuged (30,000 rpm for 60 minutes at 4°C). Finally, the supernatant was stored in microtubes at -80°C until analysis.

### Determination of superoxide dismutase activity

SOD activity was determined using an automated biochemical analyzer (Cobas Mira *Plus*) and a commercially available kit (Ransod 504; Randox Laboratories®, UK) according to the manufacturer's recommendations. SOD activity was measured as the degree of reaction inhibition. A mixed substrate, buffer, xanthine oxidase, and standard were used to calculate the SOD activity, which was normalized to the tissue weight and expressed as U/g. The method was adapted from the protocol described by Stocks et al.^27^

### Determination of glutathione peroxidase and glutathione reductase activity

GPx and GR activities were determined in a biochemical analyzer (Cobas Mira *Plus*) by using a commercially available kit (Ransel 505 and GR 2368; Randox Laboratories, UK), according to the manufacturer's recommendations. Enzyme activity was normalized to the tissue weight and expressed in U/g. These protocols were adapted from Paglia & Valentine^28^ and Melissinos et al.^29^

### Multiplex cytokine analysis

Multiplex cytokine analysis was performed by Genese Produtos Diagnósticos Ltda (São Paulo, SP, Brazil). In tissue preparation for cytokine analysis (IFN-γ, IL-4, IL-10, IL-12 [p70], IL-13 and TNF-α), frozen intestine (0.25 g) was homogenized at low speed in RIPA buffer - 50 mM Lysis Buffer (Catalog # 20-188 - Millipore®) containing Protease Inhibitor Cocktail, 50x (Cat # G6521-Promega) in a volume corresponding to 3 times the absolute mass of the sample. The samples were centrifuged for 30 seconds (3,500 rpm), and all materials were sonicated in an ice bath for 10 min. The supernatant was transferred to a new tube and centrifuged at 10,000 rpm and 4°C. After centrifugation, 200 μL of the supernatant was pipetted into an Eppendorf tube and stored at -20°C. Millipore multiscreen, 96-well filter plates (Bedford, MA, USA) were used for all multiplex cytokine kits. Assays were run in triplicate according to the manufacturer's protocols. Data were collected using the Milliplex Analyser 200 version 2.3 (Luminex, Austin, USA). Data analysis was performed using the Analyst software, version 3.1.

### Statistical analysis

The results are expressed as the mean ± standard error of the mean (SEM) and were analyzed by using one-way analysis of variance (ANOVA) with Tukey's post hoc test. Additionally, a p-value equal to or less than 0.05 was considered to be statistically significant. All statistical analyses were performed using R software. Data distribution was analyzed using the Shapiro test and histogram analysis.

## Results

### Clinical evaluation

All mice treated with TNBS showed significant inflammatory manifestations compared to those observed in the other groups. Although ethanol (a diluting agent) is known to induce colitis, it was not an aggressive agent at the concentrations used in this study (Fig. Supplement 1). There was a significant improvement in the clinical condition of animals that received TNBS plus HBO (Fig. Supplement 1). No significant differences were observed between the SALINE and ETHANOL groups.

### Body weight

**Fig. 1:**
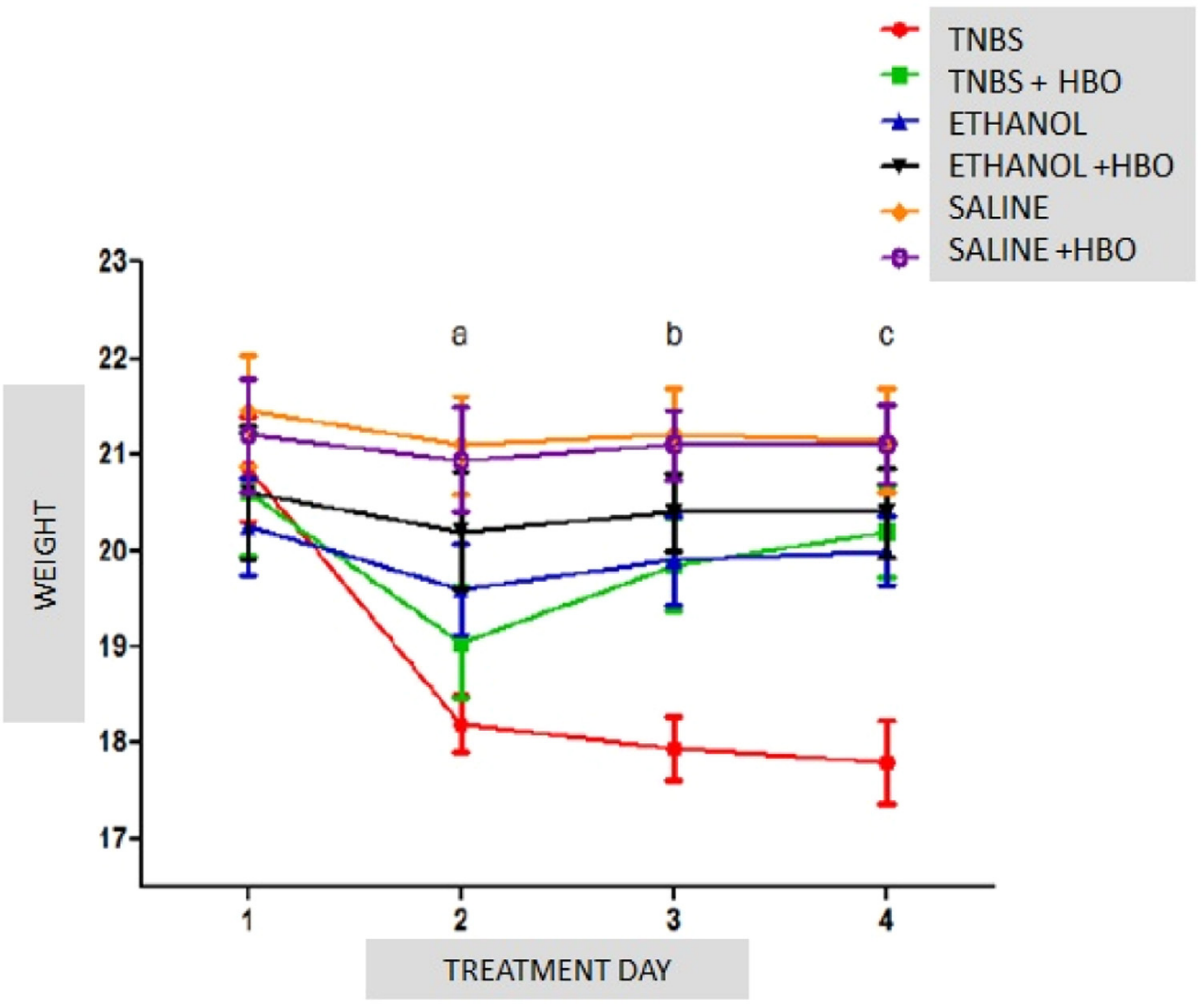
Weight variations in all groups during the experimental period. The values represent the mean ± SEM; letters indicate significant differences where a = **, ** = p < 0.005, TNBS vs. SALINE; SALINE + HBO/b = *, ** and ***, * = p ≤ 0.05, TNBS vs. TNBS + HBO and ETHANOL, ** = p < 0.005, TNBS vs. ETHANOL+HBO, *** = p < 0.0001, TNBS vs. SALINE and SALINE + HBO/c = *, ** and ***, * p ≤ 0.05, TNBS vs. ETHANOL, ** = p < 0.005, TNBS vs. TNBS+HBO and ETHANOL+HBO, *** p ≤ 0.0001, TNBS vs. SALINE and SALINE + HBO; (n = 10 animals/group).

The evaluation of treated and untreated animals within each subgroup showed a body weight loss of 12.71% in the TNBS group 24 h after the administration of the inducer. Animals treated with HBO exhibited a weight loss of 7.52% in the first 24 h, with almost complete weight recovery by the end of the experimental period. At the end of the treatment, body weight significantly decreased (14.63%) in the TNBS group, and this effect was reversed by HBO, as observed in the TNBS + HBO group, which had an average weight loss of only 1.94% at the end of the experimental period. There were no significant differences among the other groups (Fig. 1).

### Histological evaluation of colonic inflammation

**Fig. 2:**
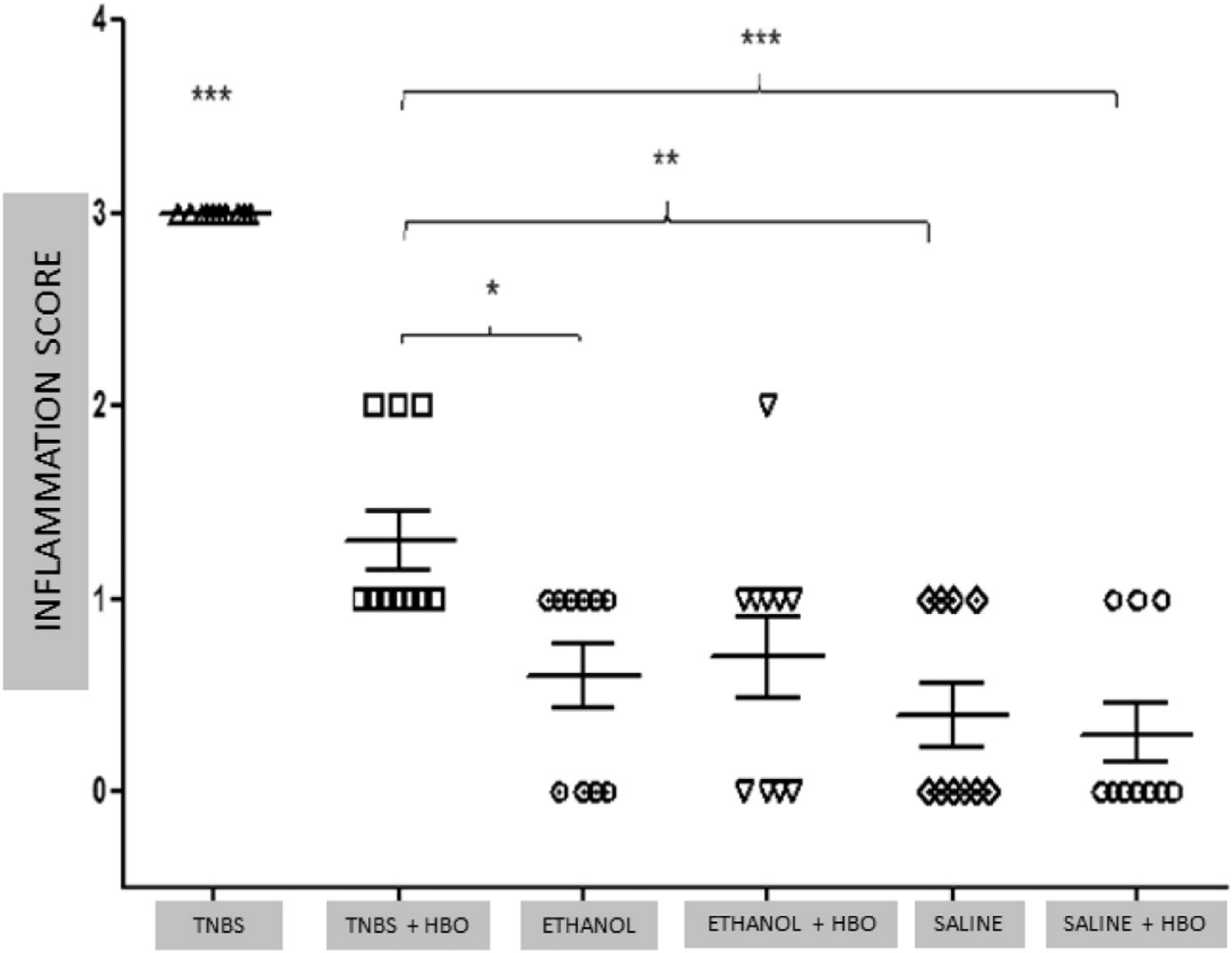
Inflammation Score ‒ Evaluation of the animals. The values represent the mean ± SEM, * = p < 0.05, TNBS + HBO vs. ETHANOL, ** = p < 0.001, TNBS + HBO vs. SALINE, *** + p < 0.0001, TNBS + HBO, ETHANOL, ETHANOL + HBO, SALINE and SALINE + HBO. (n = 10 animals/group). Macroscopic damage scale: 0 = Normal appearance; 1 = Focal ulcer; 2 = Multifocal ulcer; and 3 = Diffuse ulcer and necrosis.

Histological analysis of the intestinal tissue from the different groups showed that animals treated with TNBS had intense cell infiltration and an absence of villi in the tissue (Fig. Supplement 2A). Correspondingly, the structure was preserved in the tissues of animals treated with HBO (Fig. Supplement 2B). No differences were observed among the other groups (Fig. Supplement 2C, 2D, 2E, and 2F). The results of the inflammation histological score demonstrated a significant improvement in the TNBS + HBO group compared with the TNBS group (Fig. 2).

### Analysis of antioxidant enzymes

**Fig. 3:**
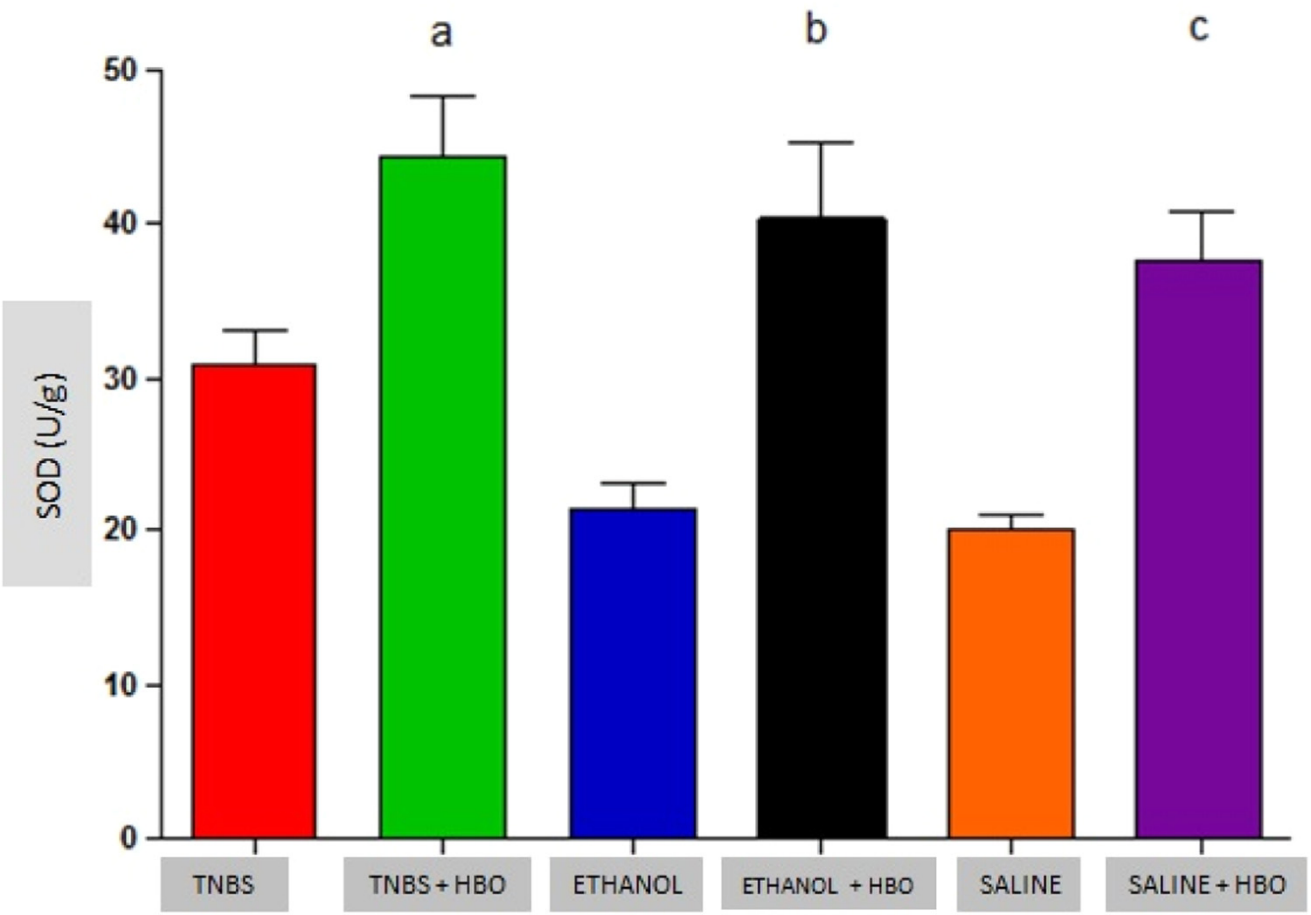
Measurements of SOD in the intestines of treated and untreated mice subjected or not subjected to HBO therapy. The values represent the mean ± SEM (n = 10 animals/group) (SOD, Superoxide Dismutase).

**Fig. 4:**
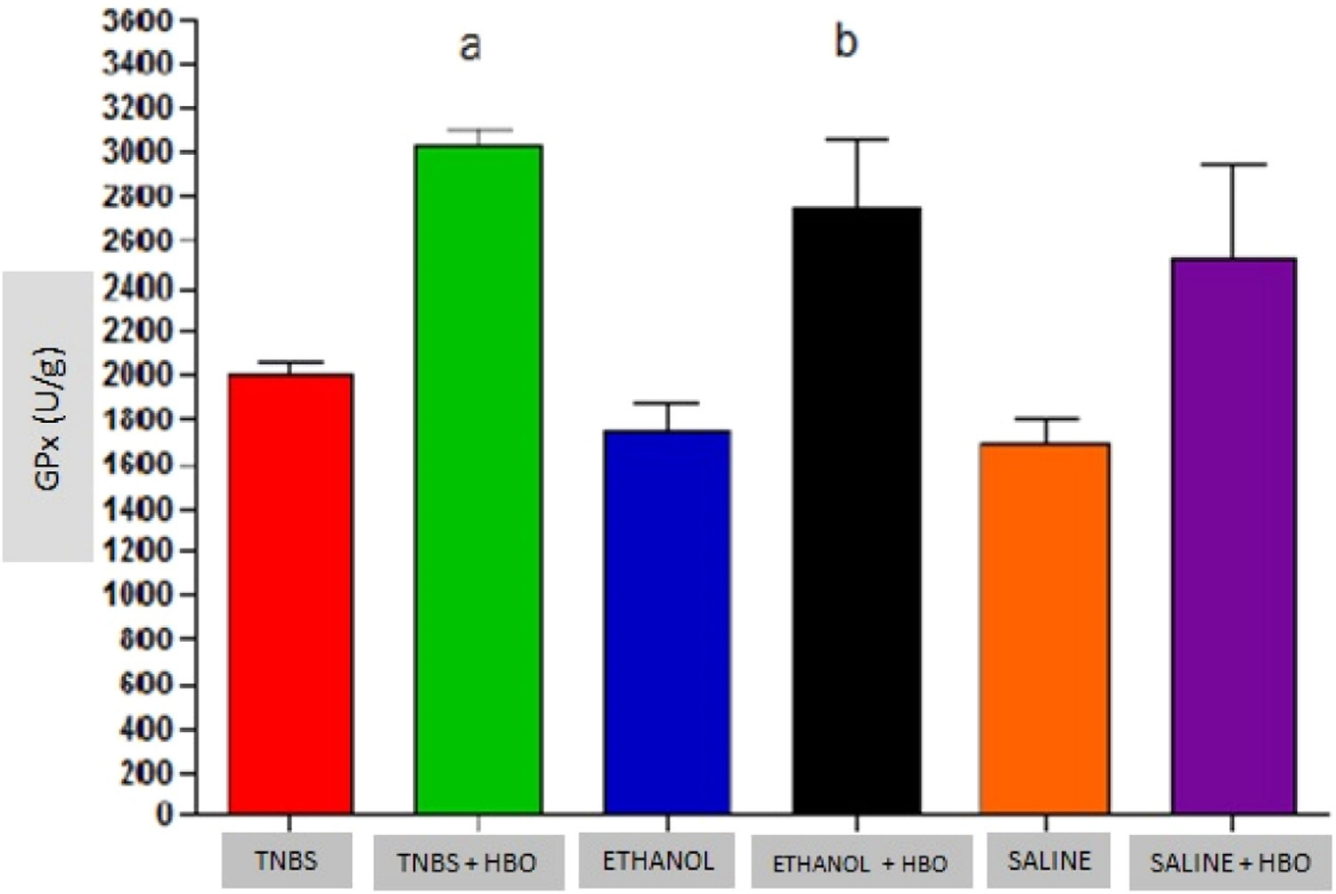
Measurements of GPx in the intestines of treated and untreated mice subjected or not subjected to HBO therapy. The values represent the mean ± SEM; letters indicate significant differences where a = *, **, * = p < 0.05, TNBS+HBO vs. TNBS; ** = p < 0.005, TNBS + HBO vs. ETHANOL and SALINE/b = *, * = p < 0.05, ETHANOL + HBO vs. ETHANOL and SALINE (n=10 animals/group) (GPx, Glutathione Peroxidase).

**Fig. 5:**
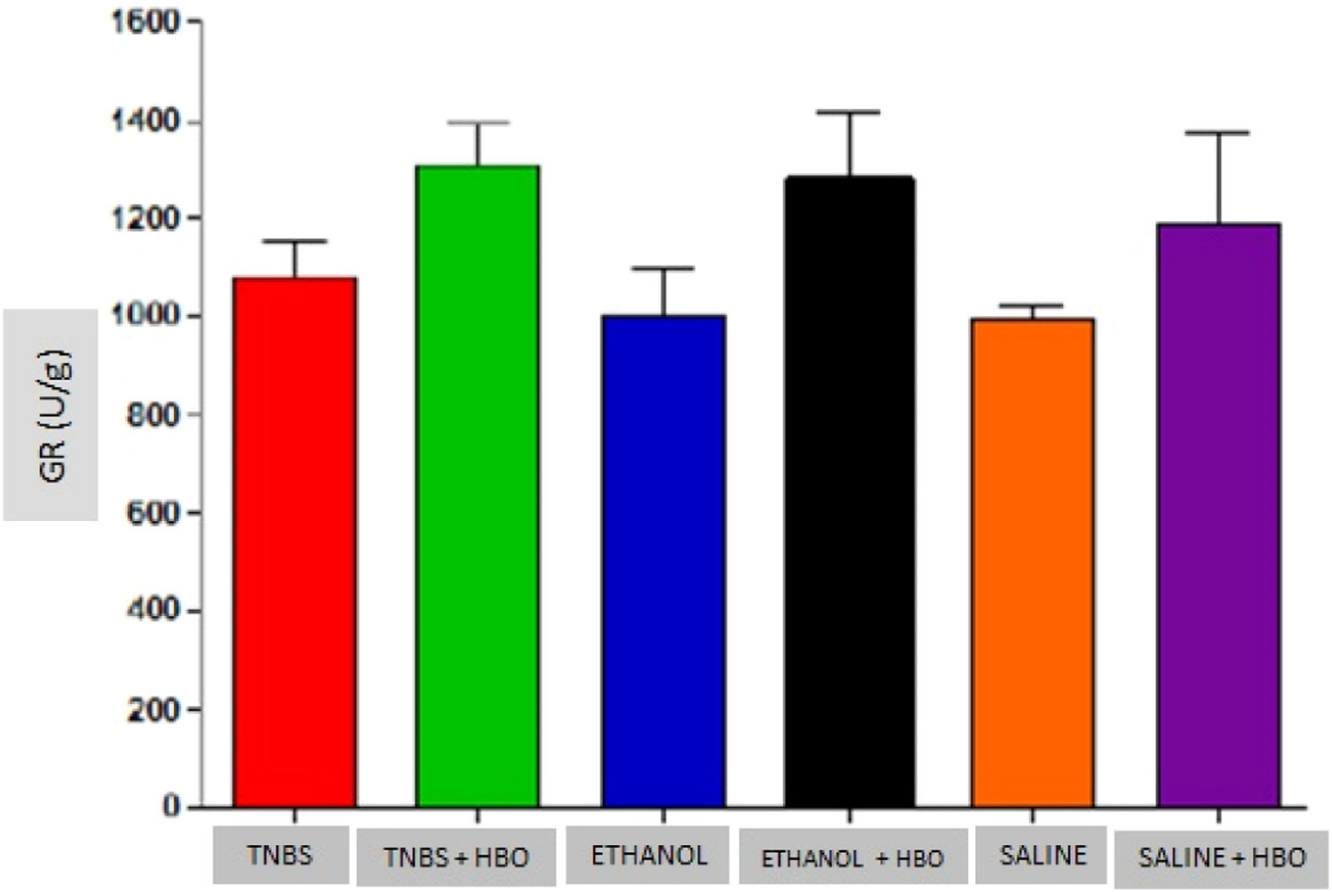
Measurements of GR in the intestines of treated and untreated mice subjected or not subjected to HBO therapy. The values represent the mean ± SEM; no differences were observed between the TNBS, ETHANOL+HBO and SALINE/TNBS+HBO, ETHANOL and ETHANOL+HBO groups (GR, Glutathione Reductase).

An increase in the activity of all antioxidant enzymes was observed in the groups that received HBO compared with the groups that did not receive HBO (Figs. 3 to 5). These results suggest that HBO modulates the activity of antioxidant enzymes that play a role in oxidative stress, regardless of colitis induction.

Animals that received TNBS exhibited increased expression of antioxidant enzymes compared to animals in the control groups; however, these changes were not statistically significant (Table 1). In animals treated with TNBS and in animals that received HBO, there was a statistically significant increase in the expression levels of SOD and GPx (Figs. 3 and 4) compared to the control groups. The same result was not observed for GR, for which there was no significant increase in activity (Fig. 5). Although the administration of TNBS resulted in increased SOD and GPx levels when compared with control individuals, the application of HBO resulted in a greater increase.

**Table 1:** Antioxidant activity in the intestines of mice.

Group	SOD (U/g)	GPx (U/g)	GR (U/g)
TNBS	31.01 ± 2.08^a^	2005.97 ± 51.57^a^	1077.33 ± 73.74^a^
TNBS+HBO	44.35 ± 4.00^b^	3028.34 ± 78.16^b^	1310.90 ± 80.04^a^
ETHANOL	21.62 ± 1.59^a^	1748.71 ± 134.24^a^	1004.90 ± 94.67^a^
ETHANOL+HBO	40.37 ± 5.00^a,b^	2741.17 ± 307.72^a,b^	1280.15 ± 137.66^a^
SALINE	20.14 ± 0.82^a^	1693.84 ± 109.38^a^	998.57 ± 22.81^a^
SALINE+HBO	37.64 ± 3.27^a,b^	2511.85 ± 429.79^a,b^	1190.97 ± 184.56^a^

### Analysis of cytokines

**Fig. 6:**
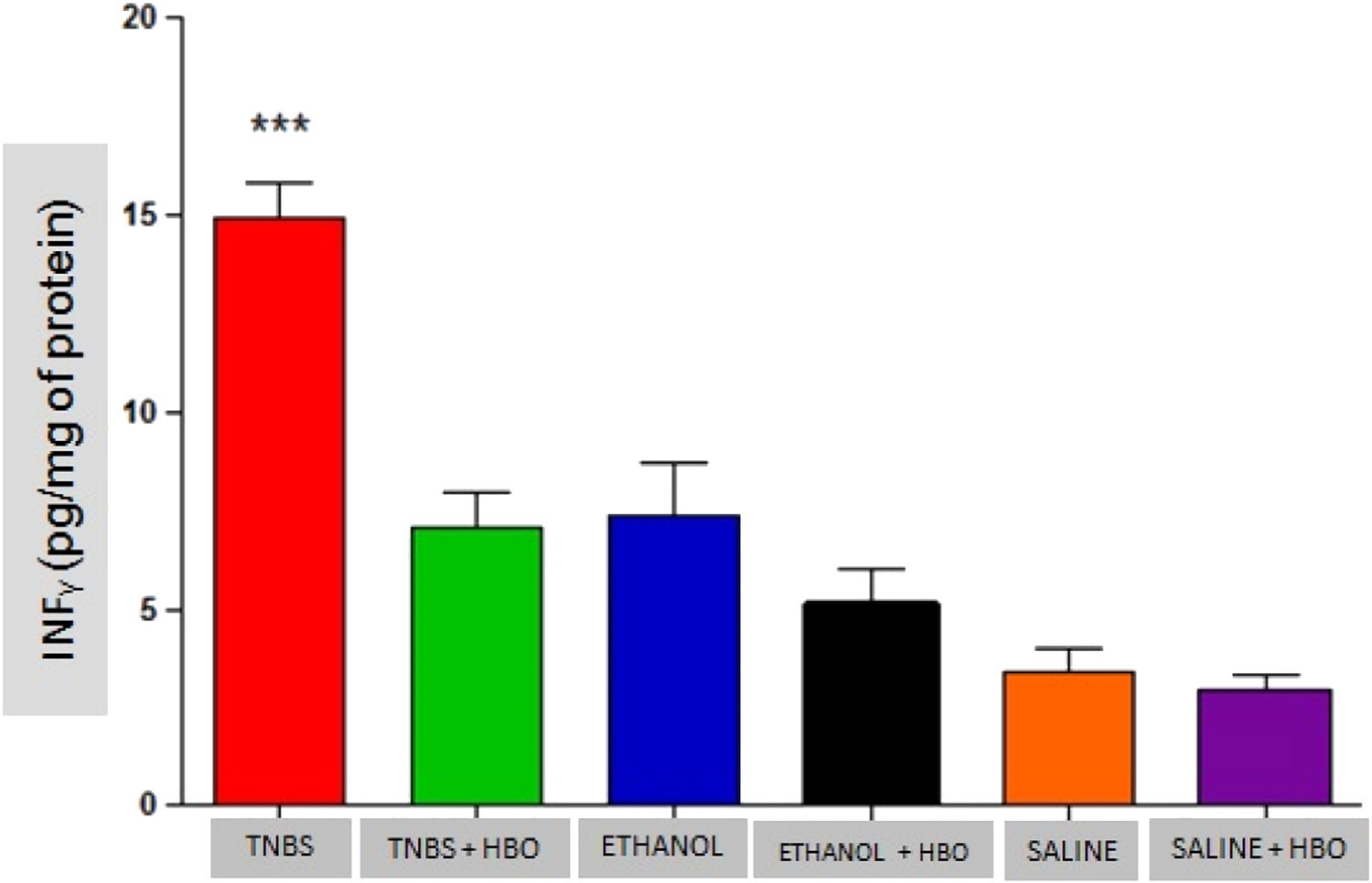
Measurements of interferon γ in the intestines of treated and untreated mice subjected or not subjected to HBO therapy. The values represent the mean ± SEM; *** = p < 0.0001 TNBS vs. TNBS+HBO, ETHANOL, ETHANOL + HBO, SALINE and SALINE + HBO (n = 10 animals/group).

**Fig. 7:**
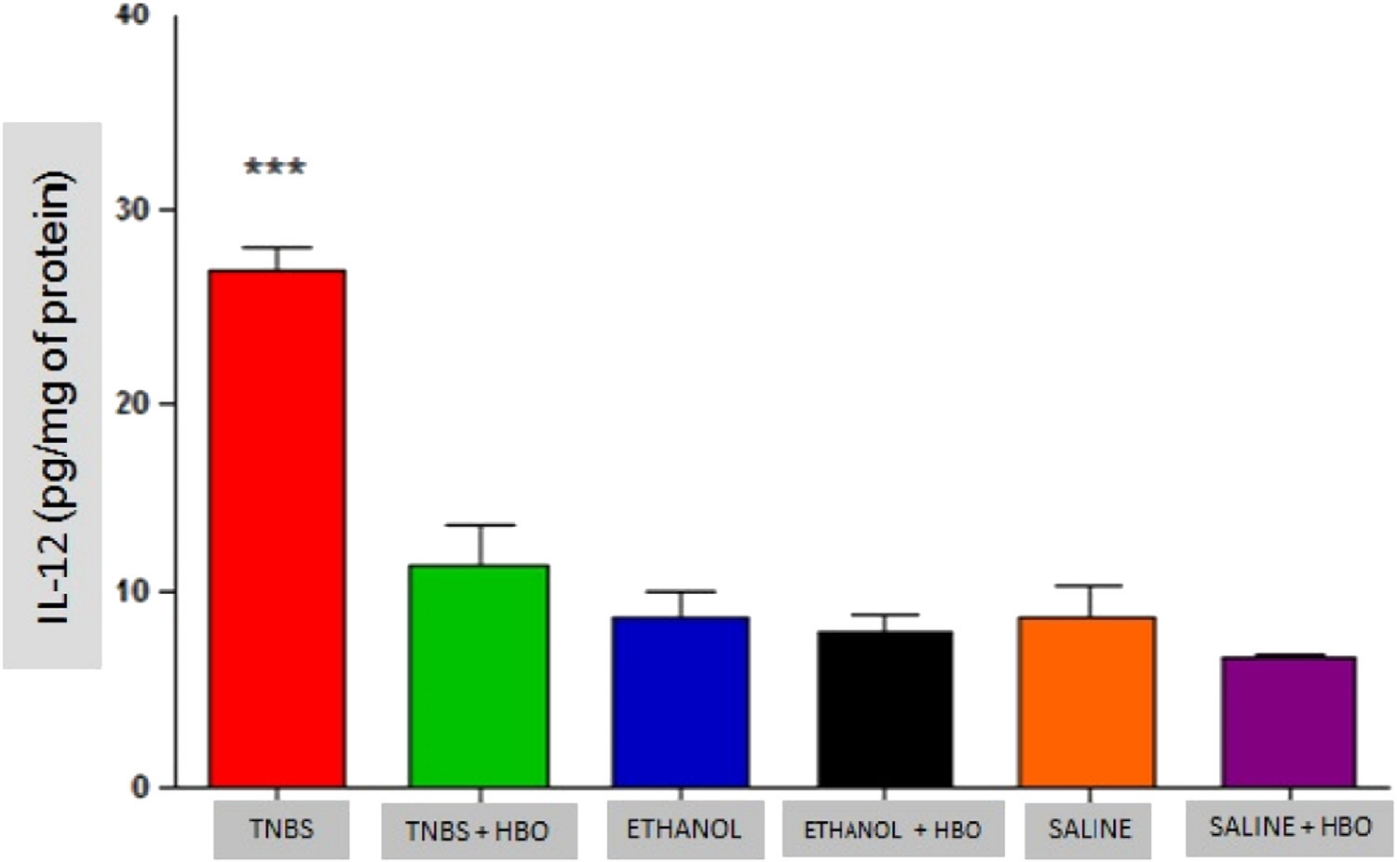
Measurements of IL-12 in the intestines of treated and untreated mice subjected or not subjected to HBO treatment. The values represent the mean ± SEM; *** = p ≤ 0.0001 TNBS vs. TNBS + HBO, ETHANOL, ETHANOL+HBO, SALINE and SALINE+HBO (n = 10 animals/group).

**Fig. 8:**
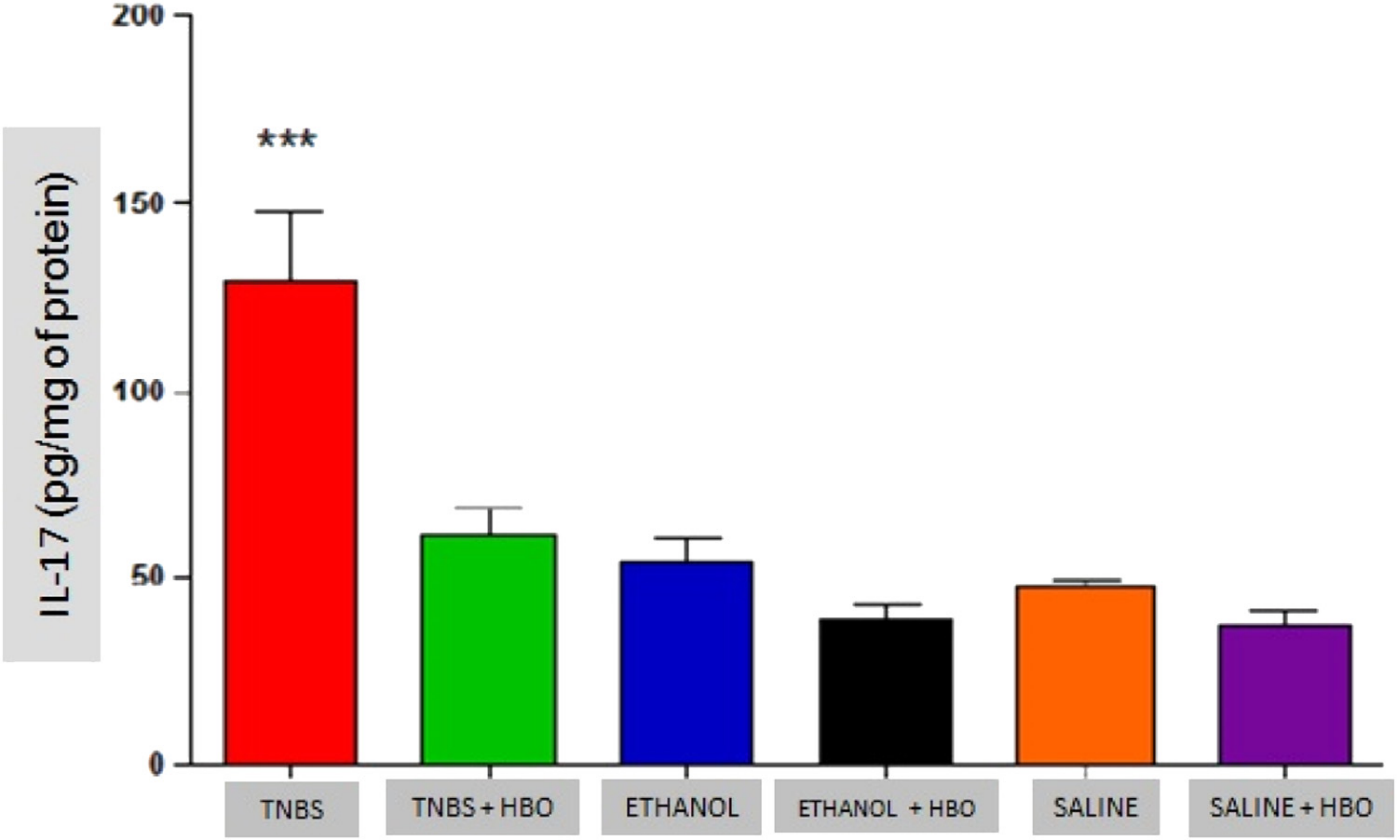
Measurements of IL-17 in the intestines of treated and untreated mice subjected or not subjected to HBO therapy. The values represent the mean ± SEM; *** = p ≤ 0.0001 TNBS vs. TNBS + HBO, ETHANOL, ETHANOL + HBO, SALINE and SALINE + HBO (n = 10 animals/group).

**Fig. 9:**
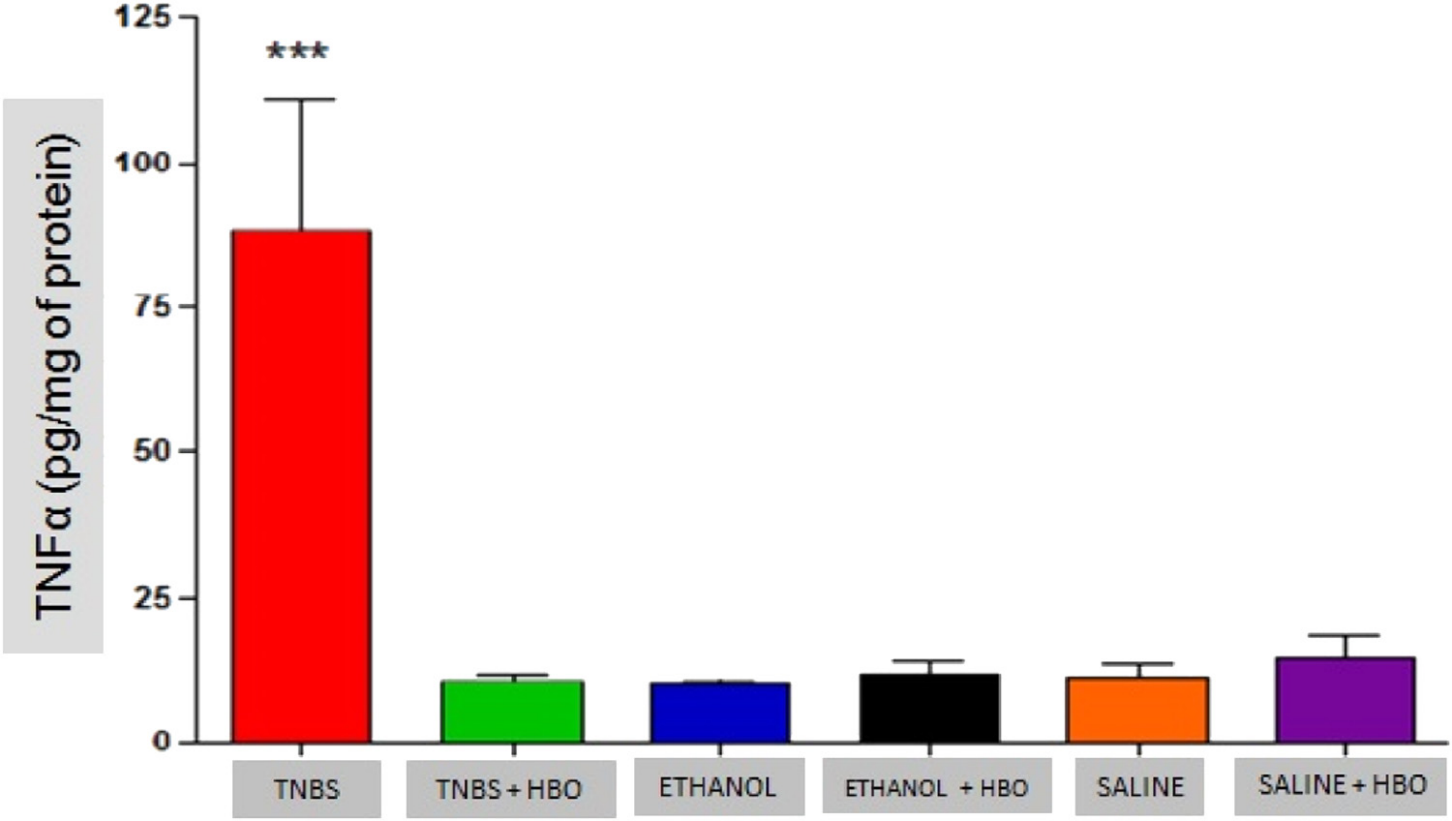
Measurements of TNF-α  in the intestines of treated and untreated mice subjected to HBO application. The values represent the mean ± SEM; *** = p ≤ 0.0001 TNBS vs. TNBS + HBO, ETHANOL, ETHANOL+HBO, SALINE and SALINE + HBO (n = 10 animals/group).

As described in the literature, animals treated with TNBS demonstrated increased production of IFN-γ, IL-12, IL-17, and TNF-α. The application of HBO reversed the elevation of these cytokines in the intestines of animals treated with TNBS, as observed in the animals in the TNBS + HBO group (Fig. 6, Fig. 7, Fig. 8, Fig. 9).

**Fig. 10:**
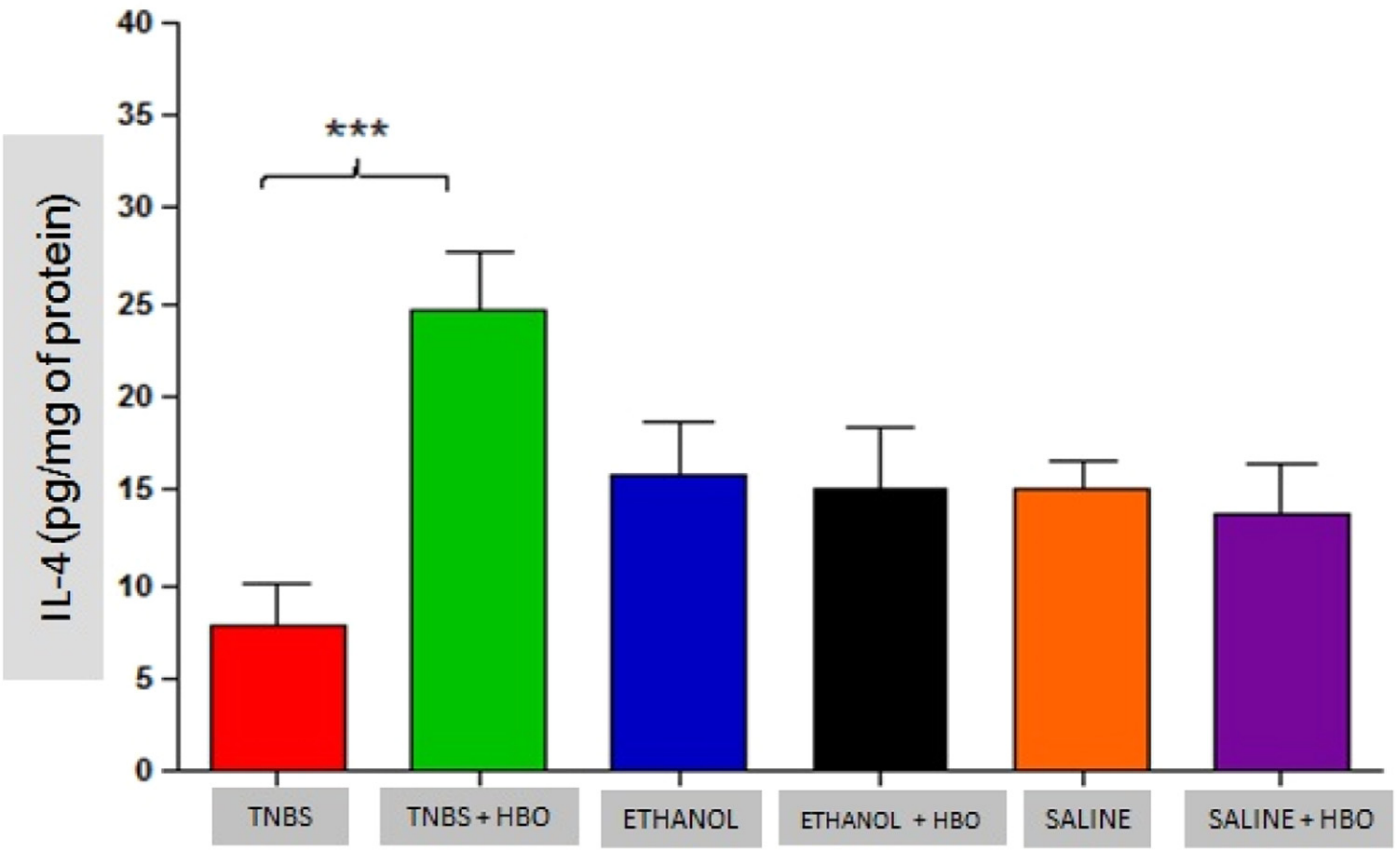
Measurements of IL-4 in the intestines of treated and untreated mice subjected or not subjected to HBO therapy. The values represent the mean ± SEM; *** = p ≤ 0.0001 TNBS vs. TNBS + HBO, ETHANOL, ETHANOL + HBO, SALINE and SALINE + HBO (n = 10 animals/group).

**Fig. 11:**
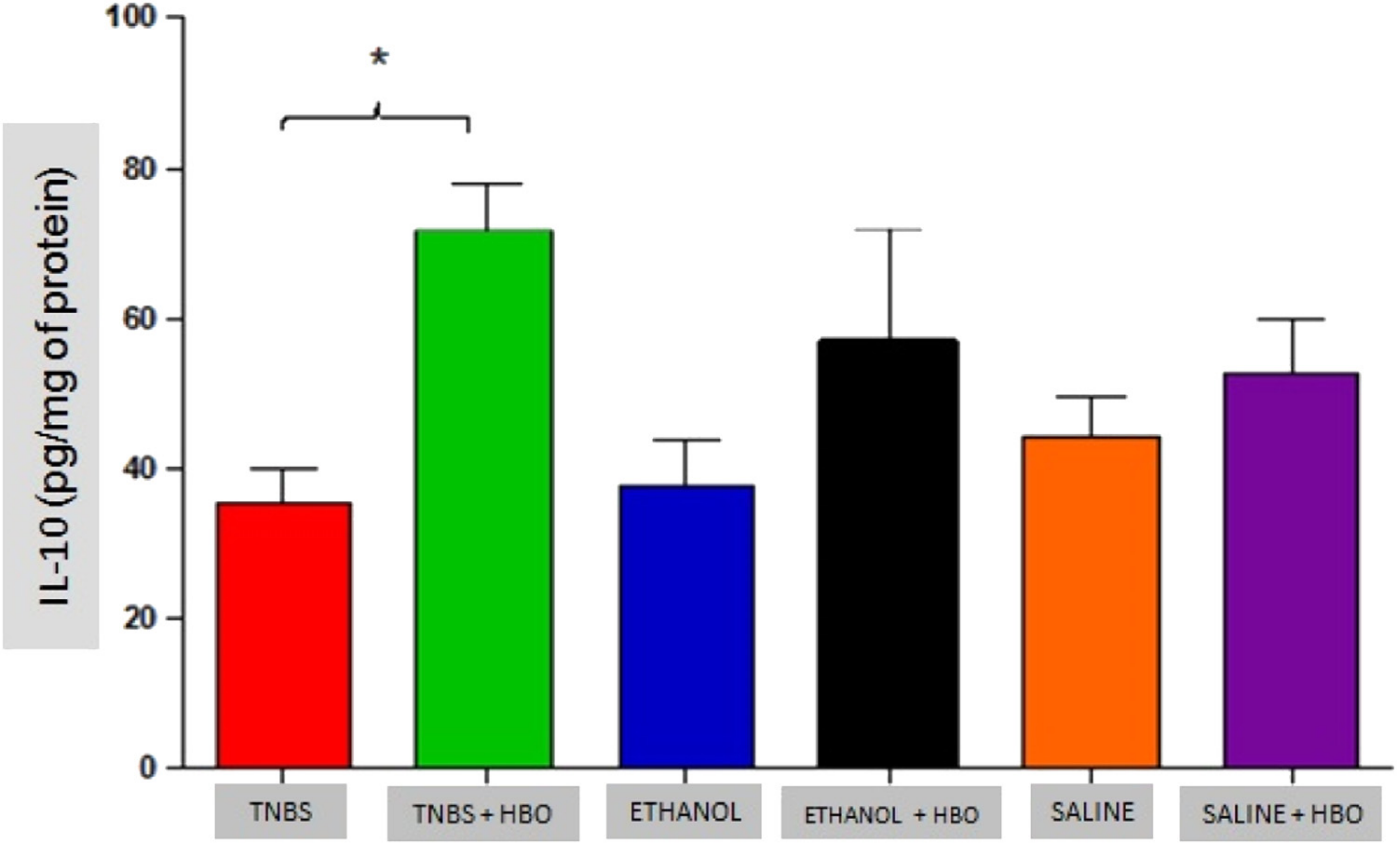
Measurements of IL-10 in the intestines of treated and untreated mice subjected or not subjected to HBO therapy. The values represent the mean ± SEM; *** = p ≤ 0.0001 TNBS vs. TNBS+HBO, ETHANOL, ETHANOL+HBO, SALINE and SALINE+HBO (n = 10 animals/group).

An increase in IL-4 levels occurred only in the TNBS + HBO group (Fig. 10) and was not observed in the other groups treated with HBO (ETHANOL + HBO and SALINE + HBO). Correspondingly, IL-10 levels increased in all groups that received HBO; however, the difference was only statistically significant when comparing the TNBS + HBO group with the TNBS group (Fig. 11). Neither TNBS nor HBO treatment promoted changes in IL-13 levels (Fig. Supplement 3).

These results suggest that HBO is associated with the regulation of the cytokine profile in the inflammatory process, thus reducing proinflammatory activity and consequently increasing anti-inflammatory activity.

## Discussion

In the present study, HBO in mice with TNBS-induced colitis resulted in clear clinical improvements as well as increases in SOD and GPx levels in animals with or without intestinal inflammation and a small (non-significant) increase in GR. Similar to the present results, previous studies have demonstrated that after HBO, there is an increase in the activity of antioxidant enzymes and a reduction in oxidative stress.^26^,^30^, ^31^, ^32^ The application of HBO is believed to increase ROS production, which can act as an anti-infective agent by eliminating the desired conditions for bacteria that lack antioxidant defense pathways in the gut.^33^ Nevertheless, the gut microbiota is radially segregated by a radial oxygen gradient, and HBO may alter mucosal adherent bacterial populations differently from those in feces.^34^ In ulcerative colitis, HBO improves disease activity by reducing STAT3-mediated neutrophil degranulation and shifting microbial composition and metabolism.^35^ Furthermore, stimulation of the antioxidant signaling pathway in the mucosa may lead to a reduction in inflammatory processes^30^,^36^,^37^ without tissue damage. However, the mechanism through which it induces the activation of these enzymes is poorly understood.

Another mechanism of action of HBO therapy that has been described in the literature is the modulation of the immune response through increased expression of growth factors and reduced expression of inflammatory cytokines.^33^ The authors also observed that animals administered with TNBS exhibited increased proinflammatory activity (IFN-γ, IL-12, IL-17, and TNF-α) and decreased anti-inflammatory activity (IL-4 and IL-10). However, after HBO, these values became inverted, a phenomenon that was not observed in the untreated animals plus HBO (ETHANOL + HBO and SALINE+HBO groups). Among the suppressed cytokines is IL-17, which is involved in the pathogenesis of IBD, and its elevation is significantly related to the development of CD.^38^,^39^ Thus, the present results show that HBO is an important modulator of IL-17 production, suggesting an anti-inflammatory action through the inhibition of the Th17 pathway. Therefore, it is possible to relate the suppression of proinflammatory cytokines (Th1 and Th17 pathways) observed in the TNBS + HBO group to an increase in anti-inflammatory cytokines (IL-10).

The present study demonstrates that HBO increases SOD and GPx levels and can reduce proinflammatory cytokines, thus improving intestinal inflammation and supporting the use of HBO in the treatment of at least some cases of IBD.

## Conclusion

The present findings showed that the application of HBO modulated the inflammatory response by increasing the activity of SOD and GPx levels, reducing pro-inflammatory cytokines (such as IFN-γ, IL-12, IL-17 and TNF-α), and increasing the anti-inflammatory cytokines IL-4 and IL-10. Moreover, there was a remission of the inflammatory process in the tissue subjected to histological evaluation and improvement in the clinical manifestations associated with the development of TNBS-induced inflammation. Taken together, these results suggest that HBO is effective in treating animals with TNBS-induced intestinal inflammation by increasing the activity of antioxidant enzymes and modulating the cytokine profile.

## Authors’ contributions

Fernanda Serafim Nakutis: Conceptualization, analysis, interpretation of data, formal analysis, visualization, writing of the original draft.

Iêda Nishitokukado: Methodology, formal analysis, visualization, writing, review and editing.

Fabiana Maria dos Santos: Methodology, formal analysis, visualization, writing, review and editing.

Carmen Lucia Ortiz-Agostinho: Methodology, formal analysis, visualization, writing, review and editing.

Daniel Teixeira de Alencar: Methodology.

Cassiana Ganem Achtschin: Methodology.

Valeria Sutti Nunes: Methodology, formal analysis.

André Zonetti Arruda Leite: Conceptualization, supervision, analysis, interpretation of data, critical review, writing, review, and editing.

Aytan Miranda Sipahi: Conceptualization, supervision, analysis, and interpretation of data, critical review, writing, review, and editing.

All authors contributed to the critical revising and the final approval of the manuscript.

## Declaration of Competing Interest

The authors declare no conflicts of interest.

## Appendix Supplementary materials

Image, application 1
