# Supplementary material for: Evaluation of oxidative stress in an experimental model of Crohn's disease treated with hyperbaric oxygen therapy
Source: Clinics (Sao Paulo). 2023 Nov 15;78:100305. doi: 10.1016/j.clinsp.2023.100305 (PMC10685139; doi:10.1016/j.clinsp.2023.100305)
Supplement: Supplementary file 1 [file mmc1.docx]

**CLINICS-D-22-0580_Supplementary Material**


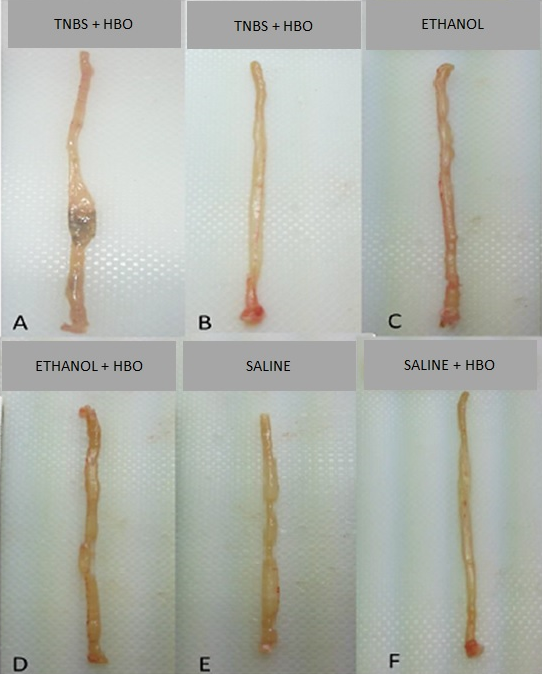


**Figure Supplement 1** Examples of intestines from the 6 groups. Macroscopic damage scale: 0 = normal appearance; 1 = focal ulcer; 2 = multifocal ulcer; and 3 = diffuse ulcer and necrosis. (1A) TNBS group - diffuse ulceration and necrosis (score of 3); (1B) TNBS+HBO group - tissue with normal appearance without apparent ulceration and/or necrosis; (1C) ETHANOL group - normal appearance; (1D) ETHANOL+HBO group - normal appearance; (1E) SALINE group - normal appearance; (1F) SALINE+HBO group - normal appearance.


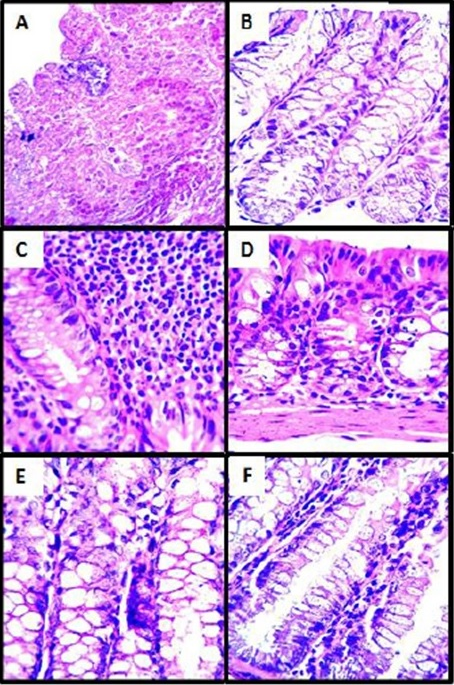


**Figure Supplement 2**  Microscopic evaluation of the intestine (H&E) in cross section (400x). (A) TNBS group: invasive colitis, with intense inflammatory infiltrates throughout the intestinal wall; (B) TNBS+HBO group: mild colitis, with preservation of villi; (C) ETHANOL group: normal histological appearance; (D) ETHANOL+HBO group: normal histological appearance; (E) SALINE group: normal histological appearance; (F) SALINE+HBO group: normal histological appearance.


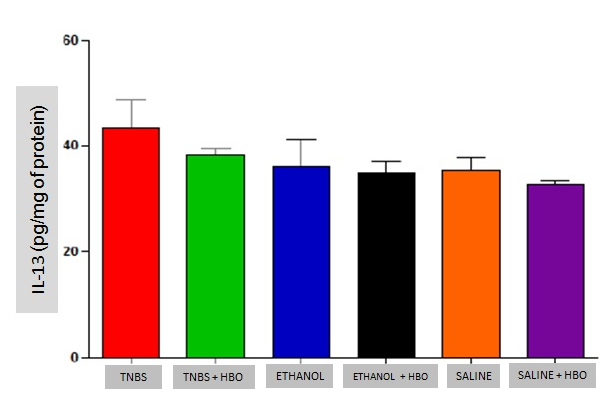


**Figure Supplement 3** Measurements of IL-13 in the intestines of treated and untreated mice subjected or not subjected to HBO therapy. The values represent the mean ± SEM; there were no differences between groups. NBBS *vs.* TNBS + HBO, ETHANOL, ETHANOL + HBO, SALINE and SALINE + HBO (n = 10 animals/group).
